# Quantitative data from the SeptiFast real-time PCR is associated with disease severity in patients with sepsis

**DOI:** 10.1186/1471-2334-14-155

**Published:** 2014-03-21

**Authors:** Ingrid Ziegler, Per Josefson, Per Olcén, Paula Mölling, Kristoffer Strålin

**Affiliations:** 1 Department of Infectious Diseases, Örebro University Hospital, S-701 85 Örebro, Sweden; 2 School of Health and Medical Sciences, Örebro University, Örebro, Sweden; 3 Department of Laboratory Medicine, Örebro University Hospital, Örebro, Sweden; 4 Department of Infectious Diseases, Karolinska University Hospital, Stockholm, Sweden

## Abstract

**Background:**

The commercial test, SeptiFast, is designed to detect DNA from bacterial and fungal pathogens in whole blood. The method has been found to be specific with a high rule-in value for the early detection of septic patients. The software automatically provides information about the identified pathogen, without quantification of the pathogen. However, it is possible to manually derive Crossing point (Cp) values, i.e. the PCR cycle at which DNA is significantly amplified. The aim of this study was to find out whether Cp values correlate to disease severity.

**Methods:**

We used a study cohort of patients with positive results from SeptiFast tests for bacteria from a recent study which included patients with suspected sepsis in the Emergency department. Cp values were compared with disease severity, classified as severe sepsis/septic shock or non-severe sepsis, according to the criteria of the American College of Chest Physicians/Society of Critical Care Medicine.

**Results:**

Ninety-four patients were included. The prevalence of severe sepsis/septic shock in the study was 29%. SeptiFast positive tests from patients with severe sepsis/septic shock had significantly lower Cp values compared with those from patients with non-severe sepsis, median 16.9 (range: 7.3 - 24.3) versus 20.9 (range: 8.5 - 25.0), p < 0.001. Positive predictive values from the SeptiFast test for identifying severe sepsis/septic shock were 34% at Cp cut-off <25.0, 35% at Cp cut-off <22.5, 50% at Cp cut-off <20.0, and 73% at Cp cut-off <17.5. Patients with a positive Septifast test with a Cp value <17.5 had significantly more severe sepsis/septic shock (73% versus 15%, p < 0.001), were more often admitted to the Intensive Care Unit (23% versus 4%, p = 0.016), had positive blood culture (BC) more frequently (100% versus 32%, p < 0.001) and had longer hospital stays (median 19.5 [range: 4 - 78] days versus 5 [range: 0 - 75] days, p < 0.001) compared with those with a Cp value >17.5.

**Conclusions:**

Our results suggest that introducing quantitative data to the SeptiFast test could be of value in assessing sepsis severity. Moreover, such data might also be useful in predicting a positive BC result.

## Background

Bloodstream infections are associated with high morbidity and mortality rates worldwide [1,2]. Prompt administration of adequate antibiotics is crucial for successfully treating severe sepsis and septic shock [3,4]. Blood culture (BC) is the current ‘gold standard’ for diagnosing bloodstream infections, but the method has several limitations. Firstly, the method has a low sensitivity both to slow-growing and fastidious organisms [5] and when antibiotics have been given prior to culture [6,7]. Previous studies have shown that a positive BC is only found in about 30% of patients with severe sepsis and septic shock [8]. Secondly, it takes between 24 to 72 hours before the pathogen is identified. By implementing new techniques, such as matrix-assisted laser desorption ionization-time of flight (MALDI-TOF), the time to pathogen identification can be significantly shortened, and it has been shown that this reduction has a significant effect on the time to adequate antibiotic treatment [9,10].

For better, more appropriate initial antibiotic treatment, techniques that can confirm the infectious aetiology as soon as possible are needed. Using polymerase chain reaction (PCR) analysis to detect bacterial DNA in blood samples provides a result within hours, and is virtually unaffected by the prior use of antibiotics [5]. Additionally, PCR enables the quantification of bacterial DNA.

For broad, routine use of PCR techniques in sepsis diagnostics, there is a need for validated commercial assays. There are already a few commercial tests available for the molecular identification of pathogens directly on blood samples [11-13]. Among such tests, the Light Cycler SeptiFast Test (Roche Diagnostics) is, to date, the most used and investigated in clinical studies [14], and it has been approved for clinical use in Europe. The method has been evaluated in a systematic review [15], and was found to be specific, with a high rule-in value for the early detection of septic patients. The SeptiFast test is used for pathogen identification, but the result does not include any quantitative information.

The diagnosis of sepsis is sometimes difficult, and for the clinician it is a major challenge to, as early as possible, identify patients at risk from septic shock and death. In addition to the clinical evaluation, laboratory tests are currently the main diagnostic tools used in the initial assessment. Thus, a rapid molecular test that can contribute to predicting the risk of developing severe sepsis or septic shock would be of significant clinical value. Previous studies have shown that a high bacterial DNA load in the blood is related to a more severe disease, an increased risk of developing septic shock, and consequently a poor prognosis [16-20]. Given this, our aim was to study whether quantitative data from the commercial SeptiFast test could inform disease severity.

For this purpose, we used a study cohort of patients with positive Septifast test results from a recent prospective study [21]. The manufacturer provided access to Crossing point (Cp) values for all positive PCR results. A Cp value represents the PCR cycle at which DNA is significantly amplified, and it is generally accepted as an indirect and inverse marker of the DNA load.

In the present study, our aim was to investigate whether the level of the Cp values could differentiate cases with severe sepsis/septic shock from those with non-severe sepsis.

## Methods

### Setting and patients

The department of Infectious Diseases, Örebro University Hospital, Sweden, serves a population of 275,000 inhabitants in the county of Örebro, and is organised into an outpatient clinic and a ward for 30 adult patients.

In the prospective study [21], all patients who were subjected to BC in the department from October 2007 to September 2008, and who gave their informed consent, were enrolled. If the patient was unable to provide consent due to illness, a next-of-kin was permitted to give the consent, or it could be given the next day. This study approach was approved by the regional ethical review board in Uppsala, Sweden. Blood samples for PCR analysis were taken at the same time as BC from all participating patients. The results of the PCR assays were unknown to the clinicians until the closure of the study. In total 1,093 patients were included in the study, and 113 positive SeptiFast PCR were found in 107 patients. In 50 of them, the same pathogen was also found in the BC. These 107 patients form the study population in the present study.

As our aim was to study bacteria, we excluded four cases which tested positive for fungi. Ten cases which were positive for coagulase-negative staphylococci (CoNS) were excluded, as they had no microbiological or clinical support for infection with CoNS, and were considered to represent skin contamination. In the end, 99 positive PCR tests from 94 patients were included in the study.

### Laboratory analyses

BC was performed with the Bactec system (Becton Dickinson and Company, Sparks, MD, USA), with a Bactec 9240 incubator, and culture duration time of 7 days. For each BC, a volume of 8–10 ml of venous blood was inoculated in one Bactec PlusAerobic/F bottle and the same volume in one Bactec Plus Anaerobic/F bottle.

Whole blood was collected in sterile EDTA tubes through the same venepuncture from which the blood samples for BC were taken. The whole blood was then stored for a maximum of 4 hrs at room temperature, or up to 3 days at +4°C, or 3 months at −70°C prior to DNA preparation. DNA was extracted manually from 1.5 mL of the EDTA blood, using the SeptiFast Prep KitMGRADE (Roche Diagnostics GmnH). At the extraction step, an internal control was added to each sample. A negative control supplied by the manufacturer was included in each extraction series, and reagent controls were used as a positive control of the PCR reactions. Quantitative PCR was performed using The SeptiFast method, which is described in details elsewhere [21]. In the SeptiFast test, the internal transcribed spacer (ITS) region is used as the target to specifically distinguish 25 different bacterial and fungal pathogens (Table 1). For the Gram-positive assay, the analysis is based on melting peaks and Cp values. To reduce false-positive results from assumed contaminants, the software includes Cp cut-off values, e.g., for *Streptococcus* species, this cut-off value is at 20 cycles, and for other Gram-positives at 25 cycles. We have no access to data from samples with Cp values above this cut-off, as the test results were then reported as negative. The assay for Gram-negative bacteria is solely based on melting peaks, and there are no Cp cut-off values. However, for test results that are positive for Gram-negatives with a Cp value above 25 cycles, the software reports only Cp >25, and provides no continuous variable. In this study, 12 cases with PCR positive for Gram-negatives with Cp values above 25 were included. In the statistical analyses we have given them a Cp value of 25.0.

**Table 1:** Bacteria and fungi detectable by the SeptiFast assay according to the manufacturer

**Gram-negative**	**Gram-positive**	**Fungi**
*Escherichia coli*	*Staphylococcus aureus*	*Candida albicans*
*Klebsiella pneumoniae*	*Staphylococcus epidermidis*	*Candida tropicalis*
*Klebsiella oxytoca*	*Staphylococcus haemolyticus*	*Candida parapsilosis*
*Serratia marcescens*	*Streptococcus pneumoniae*	*Candida krusei*
*Enterobacter cloacae*	*Streptococcus pyogenes*	*Candida glabrata*
*Enterobacter aerogenes*	*Streptococcus agalactiae*	*Aspergillus fumigatus*
*Proteus mirabilis*	*Streptococcus mitis*	
*Pseudomonas aeruginosa*	*Enterococcus faecium*	
*Acinetobacter baumannii*	*Enterococcus faecalis*	
*Stenotrophomonas maltophilia*		

### Crossing point (Cp)

During PCR, the amount of PCR product formed is measured at each cycle and reported in fluorescence units. In the SeptiFast test, this starts after cycle six, as the first cycles only focus on annealing at different temperatures. The more target DNA present in a sample, the quicker a significant PCR product is generated. A sample is positive if the amount of fluorescence produced rises above a defined threshold level. At that point, a crossing point (Cp) value is created, showing how many PCR cycles are required to reach the threshold level. Thus, the more target DNA present in a sample, the lower the Cp value will be. The Cp value is the term used in LightCycler, and is the same as the threshold cycle (Ct) value in other PCR assays.

### Clinical data and definitions

A retrospective chart review was performed by a specialist in Infectious Diseases (I.Z.) to evaluate the severity of illness, without prior knowledge of the SeptiFast data. Data collected included demographic details, underlying diseases, source of infection, antibiotic treatment, length of hospital stay, intensive care unit (ICU) admission and mortality. Laboratory parameters and clinical observations (heart rate, blood pressure, temperature, respiratory rate, pulse oximetry) from the time of admission were recorded. The patient’s clinical condition on admission was classified as systemic inflammatory response syndrome (SIRS), sepsis, severe sepsis or septic shock by using the criteria published by the American College of Chest Physicians/Society of Critical Care Medicine (ACCP/SCCM) [22], see Table 2.

**Table 2:** SIRS and Sepsis Definition (ACCP/SCCM criteria)

**SIRS systemic inflammatory response syndrome**	**Sepsis**	**Severe sepsis**	**Septic shock**
Two or more of the following criteria:	Documented infection together with 2 or more SIRS criteria.	Sepsis-induced tissue hypoperfusion or organ dysfunction, including hypotension, lactic acidosis, oliguria, hypoxemia, coagulation disorders and acute alteration in mental status.	Sepsis with hypotension, despite adequate fluid resuscitation, along with the presence of perfusion abnormalities.
• Temperature > 38°C or <36°C			
• Heart rate > 90/min			
• Respiratory rate > 20/min or PaCO _2_ < 32 mmHg (4.3 kPa)			
• WBC > 12 × 10^9^ /L, < 4 × 10^9^/L, or > 10% immature forms			

**Statistics** Medians and ranges were used for descriptive statistics of continuous variables, and percentages for categorical variables. Comparison of groups was performed using the Mann–Whitney-U test for continuous variables and the Chi-square or Fischer’s exact test for categorical variables. A p-value of < 0.05 was regarded as significant. Positive predictive values were calculated from cross-tabulations. The IBM SPSS Statistics, version 21, New York, USA, was used for calculations.

## Results

### Study population

In Table 3, demographic data and clinical characteristics are shown for the 94 patients with positive SeptiFast PCR. The main foci of infection were the urinary tract (n = 27, 29%), the gastrointestinal/bilary tract (n = 11, 12%), the skin (n = 11, 12%), orthopaedic (n = 10, 11%) and the chest (n = 9, 10%). Six (6%) patients had an unknown focus of sepsis and 15 (16%) had no evidence of bacterial infection.

**Table 3:** Demographic and clinical characteristics of the patients with a positive SeptiFast test

	**All patients (n=94)**	**Patients with Cp >17.5 (n=72)**	**Patients with Cp <17.5 (n=22)**	**P-value for comparison**
Female	35 (37%)	26 (36%)	9 (41%)	0.80
Age, years	74 (14-96)	69 (25-96)	74 (14-93)	0.36
*Co-morbidities*				
Heart disease	22 (23%)	14 (20%)	8 (36%)	0.12
Neurological disease	13 (14%)	8 (11%)	5 (23%)	0.18
Chronic renal failure	12 (13%)	10 (14%)	2 (9%)	0.54
Neoplasms	17 (18%)	14 (20%)	3 (14%)	0.50
*Clinical data*				
Antibiotics before analyses	20 (21%)	15 (21%)	5 (23%)	0.85
Blood culture positivity	45 (48%)	23 (32%)	22 (100%)	<0.001
C-reactive protein	108 (1-446)	93 (1-372)	212 (12-446)	0.002
Body temperature, C°	38.7 (37.0-41.4)	38.7 (37.1-41.0)	38.8 (37.0-41.4)	0.89
> 2 SIRS criteria	82 (87%)	61 (85%)	21 (96%)	0.19
Severe sepsis/septic shock	27 (29%)	11(15%)	16 (73%)	<0.001
Septic shock	3 (3%)	0	3 (14%)	0.01
Hospitalisation length, days	7 (0-78)	5 (0-75)	19.5 (4-78)	<0.001
Intensive care unit admission	8 (9%)	3 (4%)	5 (23%)	0.016
1 month mortality rate	8 (9%)	4 (6%)	4 (18%)	0.084

Data are presented as numbers (percentages) for categorical variables and as median values (ranges) for continuous variables.

The pathogens most frequently detected with the SeptiFast test in the study were *Escherichia coli* (n = 33), *Staphylococcus aureus* (n = 24), *Streptococcus* species (n = 13) and *Klebsiella pnemoniae/oxytoca* (n = 13). Four patients were PCR positive for more than one pathogen (Table 4). Noteworthy is that only 2 cases with *Streptococcus pneumoniae* were detected. This is due to the SeptiFast test’s very low sensitivity to *S. pneumoniae* in the prospective study, only 12% (PCR positive/BC positive =2/16) [21].

**Table 4:** Patients with a positive SeptiFast test for more than one pathogen

**Sex, age**	**Focus of infection**	**PCR result with Crossing point (Cp) values**	**Blood culture result**	**Severe sepsis/septic shock**
Male, 93 years	Cholangitis	*Escherichia coli* (Cp:13.69), *Klebsiella pneumoniae/oxytoca* (Cp:11.20)	*Escherichia coli*, *Klebsiella pneumoniae/oxytoca*	Yes
Male, 89 years	Abdominal infection	*Escherichia coli* (Cp:13.95), *Klebsiella pneumoniae/oxytoca* (Cp:17.01) *Streptococcus* species (Cp:16.67)	*Escherichia coli, Klebsiella pneumoniae/oxytoca, Streptococcus* species	Yes
Male, 81 years	Cholangitis	*Escherichia coli* (Cp:22.61), *Klebsiella pneumoniae/oxytoca* (Cp:21.48)	Negative	No
Female, 54 years	Intestinal ulceration	*Enterobacter cloacae* (Cp:8.47), *Enterococcus faecalis* (Cp:8.76)	*Enterobacter cloacae, Enterococcus faecalis*	No

Of all 94 patients with positive SeptiFast PCR, 27 patients had severe sepsis or septic shock upon arrival, according to the criteria of ACCP/SCCM. Thirty out of 99 positive PCR results were found in patients with severe sepsis/septic shock.

### Cp values in relation to severity of disease

An overview of all bacteria found by SeptiFast PCR in the study, divided into samples from patients with severe sepsis/septic shock or non-severe sepsis, is shown in Table 5. The Cp values were significantly lower in the group with severe sepsis/septic shock compared with the group with non-severe sepsis in the study population. This was also shown in the sub-groups with positive PCR test for S*. aureus*, *Streptococcus* species and *E. coli* (Table 5).

**Table 5:** Number of positive samples and median Crossing point (Cp) values of all bacteria detected by Septifast PCR, grouped into severe sepsis/septic shock and non-severe sepsis cases

**Species identified by Septifast**	**Severe sepsis/septic shock**		**Non severe sepsis**		**P-value for comparison between groups**
	**No. of cases**	**Cp median (range)**	**No. of cases**	**Cp median (range)**	
Gram positive bacteria	17	16.7 (7.3-21.7)	25	19.9 (8.8-22.2)	0.007
*Staphylococcus aureus*	8	16.8 (10.4-21.7)	16	20.3 (13.0-22.2)	0.045
*Streptococcus* species^a^	5	16.6 (14.7-16.8)	8	18.5 (17.0-19.9)	0.002
*Enterococcus faecalis*	2	18.1 (17.1-19.2)	1	8.8	NR^b^
*Streptococcus pneumoniae*	2	13.5 (7.3-19.8)	0		NR
Gram negative bacteria	13	17.1 **(**11.2-24.3)	44	21.9 (8.5-25.0)	0.004
*Escherichia coli*	8	16.4 (13.7-22.6)	25	21.0 (17.8-25.0)	0.006
*Klebsiella pneumoniae/oxytoca*	2	14.1 (11.2-17.0)	11	22.8 (20.9-25.0)	NR
*Enterobacter cloacae/aerogenes*	2	23.6 (23.0-24.3)	3	20.5 (8.5-25.0)	NR
*Pseudomonas aeruginosa*	1	21.6	4	23.2 (19.2-25.0)	NR
*Serratia marcescens*	0		1	23.3	NR
Total	30	16.9 (7.3-24.3)	69	20.9 (8.5-25.0)	<0.001

Comparison of median Cp values between groups.

^a^*Streptococcus pyogenes, S. agalactiae* and *S. mitis*^b^Not relevant, due to small numbers.

**Figure 1:**
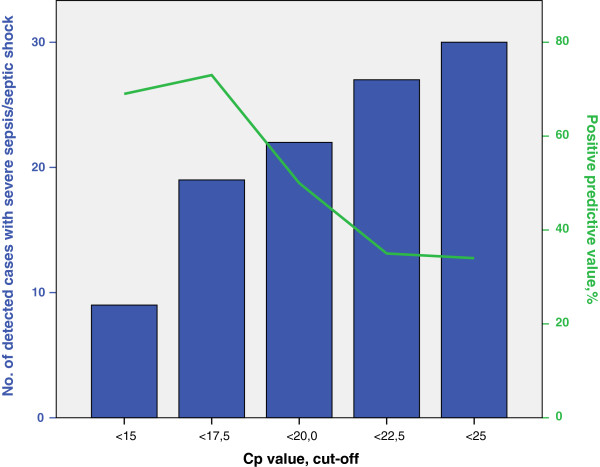
**Number of samples from patients with severe sepsis/septic shock in relation to positive predictive values.** Bars in blue show cumulative numbers of SeptiFast positive samples from patients with severe sepsis/septic shock in the entire study population at different Cp cut-off values. The green line shows positive predictive values for detection of severe sepsis/septic shock at different Cp cut-off values.

Tables 6 and 7 demonstrate positive predictive values (PPV) with different cut-off limits for the detection of severe sepsis/septic shock for the most frequently found pathogens and for all Gram-positive and Gram-negative bacteria. The positive predictive values of the SeptiFast test for detection of severe sepsis/septic shock in the entire study population are illustrated in Figure 1, and were 34% at SeptiFast Cp cut-off <25, 35% at Cp cut-off <22.5, 50% at Cp cut-off <20, and 73% at Cp cut-off <17.5.

**Table 6:** Positive predictive values of Crossing point (Cp) values with different cut-off limits for detection of severe sepsis/septic shock, for blood samples with a positive SeptiFast test for Gram-positive bacteria

**Cut-off Cp value**	***Staphylococcus aureus *** **(n = 24)**	***Streptococcus species *** **(n = 13)**	Gram-positive bacteria, other* (n = 5)	**Gram-positive bacteria total (n = 42)**
<15.0	60% (3/5)	100% (1/1)	50% (1/2)	62% (5/8)
<17.5	62% (5/8)	71% (5/7)	67% (2/3)	67% (12/18)
<20.0	55% (6/11)	38% (5/13)	80% (4/5)	52% (15/29)
<22.5	33% (8/24)			41% (17/42)

The Septifast method has a Cp cut-off at 20 cycles for test positivity for Streptococcus species, and at 25 cycles for other Gram-positive bacteria.

Data is presented as percentages (No. of SeptiFast positive samples from patients with severe sepsis /No. of SeptiFast positive samples in total, according to cut-off).

*Including: *Enterococcus faecalis* (n = 3) and *Streptococcus pneumonia* (n = 2).

**Table 7:** Positive predictive values of Crossing point (Cp) values with different cut-off limits for detection of severe sepsis/septic shock, for blood samples with a positive SeptiFast test for Gram-negative bacteria

**Cut-off Cp value**	***Escherichia coli *** **(n = 33)**	***Klebsiella pneumoniae/oxytoca *** **(n = 13)**	Gram-negative bacteria, other* (n = 11)	**Gram-negative bacteria total (n = 57)**
<15.0	100% (3/3)	100% (1/1)	0% (0/1)	80% (4/5)
<17.5	100% (5/5)	100% (2/2)	0% (0/1)	88% (7/8)
<20.0	37% (5/11)	100% (2/2)	0% (0/2)	47% (7/15)
<22.5	30% (7/23)	29% (2/7)	20% (1/5)	29% (10/35)
<25.0	30% (8/27)	22% (2/9)	33% (3/9)	29% (13/45)
>25	24% (8/33)	15% (2/13)	27% (3/11)	23% (13/57)

For samples that were positive for Gram-negative bacteria with Cp >25 (n = 12) the result is presented as >25 by the software, and not with continuous variables.

Data is presented as percentages (No. of SeptiFast positive samples from patients with severe sepsis /No. of SeptiFast positive samples in total, according to cut-off).

*Including: *Enterobacter cloacae* (n = 5), *Pseudomonas aeruginosa* (n = 5)*,* and *Serratia marcescens* (n = 1).

In Table 3, clinical characteristics for patients with Cp <17.5 and >17.5 are presented separately. Patients with Cp <17.5 do not differ in demographics compared to those with Cp > 17.5, but have significantly more severe sepsis, develop septic shock more often, require ICU treatment more often, and have longer hospital stays. We have not calculated negative predictive values (NPV), sensitivities and specificities, as we have no data on the number of patients with severe sepsis/septic shock and negative SeptiFast tests.

Eight patients with severe sepsis/septic shock attended the ICU with a median Cp value of 16.3 (range: 7.3 - 25.0). Among them, 3 patients were given advanced intensive care, including inotropic drugs and mechanical ventilation; all three were BC positive. One of them, an elderly man with spondylitis and a SeptiFast test that was positive for S*. aureus,* with a Cp value of 14.6, died. The other two patients survived; a young girl with an epidural abscess and a SeptiFast test that was positive for *Streptococcus* species, Cp 16.6, and an elderly lady with a lung abscess and a SeptiFast test which was positive for *S. pneumoniae*, Cp 7.3.

Among the 24 patients where *S. aureus* was found in PCR, four patients had endocarditis, all of them were BC positive and two of them had severe sepsis/septic shock. The median Cp value for patients with endocarditis was 13.6 (range: 10.4 - 18.6), compared with 20.2 (range: 12.1 - 22.2) for the other cases with positive *S. aureus* PCR (p = 0.007).

### BC results in relation to severity of disease and Cp values

The frequency of severe sepsis/septic shock was higher among BC positive cases, 47% (23/49) compared with BC negative ones, 14% (7/50) throughout the entire study cohort (p < 0.001).

Median Cp values were lower in samples from patients with positive BC compared with those from BC negative patients: 17.2 (range: 7.3 - 25) versus 21.5 (range: 18.6 - 25.0) throughout the entire study cohort (p < 0.001); 16.4 (range: 10.4 - 21.7) vs 20.9 (range: 20.0 - 22.2) in samples positive for *S. aureus* (p < 0.001); 16.9 (range: 14.7 - 19.9) vs 19.6 (range: 18.6 - 19.8) in samples positive for *Streptococcus* species (p = 0.049); and 19.6 (range: 13.7 - 25) vs 21.9 (range: 19.8 - 25.0) in samples positive for *E. coli* (p = 0.008).

## Discussion

To our knowledge, this is the first study which evaluates quantitative data from a commercial PCR assay for bacterial species in blood samples.

The results of the study indicate that SeptiFast Cp values do correlate to disease severity in community onset bloodstream infections. When the SeptiFast test is used for pathogen identification and PCR positivity is reported, Cp values can probably be useful in identifying patients with severe sepsis/septic shock at an early stage of the disease. A low Cp value, with a cut-off level at 17.5 cycles as proposed in this study, might be used effectively as a predictive indicator in the clinical assessment. In addition to providing rapid information about the aetiology, a positive Septifast test with a Cp value <17.5 could be an early signal of the risk for a more severe disease and indicate that the BC will probably also be positive later on.

Concordance between BC and SeptiFast PCR has been shown to be moderate [21,23-27], with a high frequency of PCR positive results not being confirmed by a positive BC, and vice versa. A positive PCR but a negative BC can occur due to fastidious microbes, sub-optimal culture conditions or antibiotic treatment before sampling. False-positive PCR results can occur due to contamination from sampling tubes or reagents. To minimise such risk, the EDTA tubes used in the study were endotoxin and DNA-free, and high-quality PCR reagents, free of bacterial or fungal DNA, were used in the SeptiFast test, as recommended by the manufacturer.

PCR positivity, even when BC remains negative, was recently found to correlate to disease severity [27]. This suggests that such a result probably often represents a relevant bloodstream infection that BC may have failed to detect.

In the present study there were several positive PCR tests with high Cp values and without microbiological verification, and it is difficult to determine their clinical significance. Many of the BC negative cases had other microbiological or clinical support, but not all of them [21].

A study cohort where all PCR positive samples were supported by a positive BC, or other microbiological or clinical verification, would have been optimal.

One possible explanation for a negative PCR despite a positive BC might be that the sample volume of approximately one mL, compared with 20 mL in BC, is not always sufficient to detect bacterial DNA, when the bacterial load is low. This is a limitation with SeptiFast and other PCR assays for bacterial identification directly on blood samples, leading to suboptimal sensitivities. Other reasons for a false-negative SeptiFast test might be competition for the PCR by human DNA in the blood, problems with the selection of species-specific targets in the test, or, for Gram-positive bacteria, especially *Streptococcus* species, a detection level set too high.

The most apparent advantage of molecular methods compared with BC is the possibility of a rapid diagnosis of aetiology with results available within hours. The time required to conduct a SeptiFast analysis is about 6 hours, but this assumes optimal laboratory conditions, which is difficult to obtain in everyday clinical practice.

A disadvantage with PCR techniques compared with BC is the lack of drug susceptibility testing. However, genetic determinants of drug resistance can be found by PCR. SeptiFast has an optional step where *S. aureus* positive samples are tested for the presence of the *mecA* gene in a subsequent run. We did not carry out this step in our study as the frequency of methicillin resistant *S. aureus* (MRSA) is low in Sweden, and all BC positive cases with *S. aureus* were methicillin sensitive in the present study.

Today, little is known about the bacterial DNA load in *S. aureus* bacteraemia [19]. In terms of the focus of *S. aureus* bloodstream infection, a correlation has been shown between time to positivity in BC, used as a surrogate marker for bacterial load, and endovascular infection sources, such as endocarditis [28]. It was recently shown that a persistently positive SeptiFast test can predict endovascular complications in patients with *S. aureus* bloodstream infections [29]. The endocarditis group in our study was very small, only four patients. Despite this, a correlation was found showing that Cp values seem to be lower in endocarditis than in other *S. aureus* bloodstream infections. Such correlation might be of significant clinical value as there is always a considerable risk of endocarditis in *S. aureus* blood stream infections. The Cp value could indicate when endocarditis should be suspected, and help distinguish which patients should be subjected to further diagnostics.

Our study has several limitations, the most important of which is the limited size of the study cohort. Further studies are needed to confirm the correlation between Cp values and disease severity, preferably with larger study cohorts and a higher proportion of patients with severe sepsis/septic shock. For certain pathogens the sample size was too small to enable any statistical analyses for the specific species. We have chosen to present results for the largest groups of specific pathogens, for Gram-positive and Gram-negative pathogens, and for the entire study cohort. The manufacturer of the SeptiFast test uses different Cp cut-offs for different pathogens, and pathogenicity and growth manner differ between species. Consequently, the associations we found for groups of pathogens put together are less reliable than the pathogen-specific associations.

The positive predictive values are calculated based on this study cohort, where the prevalence of severe sepsis was about 30%, and cannot be generalised. The suggested cut-off level at 17.5 cycles is calculated on this specific study population and needs further validation in other studies before it can be applicable in a clinical context. This value is also lower compared to other real-time PCR tests since the fluorescence in the SeptiFast test is not measured during the first six cycles.

The Cp values of the SeptiFast test can only serve as a positive predictive tool, as high Cp values cannot rule out severe sepsis/septic shock. In a real scenario, there would certainly be a number of cases with severe sepsis/septic shock and negative PCR, due to the limited sensitivity of the SeptiFast test [21]. As we lack a control group with such patients, we have not been able to calculate negative predictive values, which is a limitation.

Concerning the 12 samples that were positive for Gram-negative bacteria with Cp values >25, we unfortunately have no access to continuous variables. We have given them a Cp value of 25.0, which is a statistical concern. However, this should not affect the associations found, as data is presented in medians, and all calculations compare median values and not means.

## Conclusion

Studies have shown that PCR identification using the SeptiFast test has the potential to become a cost-effective component in managing sepsis [30-32]. The possible benefits of a more rapid diagnosis of sepsis aetiology would be earlier adequate antimicrobial treatment and reduced mortality. However, there remains a need to optimise the commercial assays for this purpose, where the most important issue would be to obtain better sensitivities. In addition, we emphasise the need for further evaluation of quantitative data and its clinical implications.

Today the results of commercial PCR tests for sepsis diagnostics do not give information about the amount of DNA of the identified pathogen. Our results indicate that such quantification data would provide valuable information to the user concerning the clinical significance of a positive test. As an improvement of such tests, we suggest introducing automatic presentation of Cp values (or Ct values when other PCR platforms than the LightCycler are used) as a part of the test result. The manufacturer of The SeptiFast test can easily derive Cp values, and it should be possible to routinely share this information with the user. It would be even better in terms of easier interpretation, if a calculation of the bacterial DNA load, measured in copies/mL, was included in the test result.

## Competing interests

Reagents and means for technical assistance were provided by Roche Diagnostics.

All authors declare no conflicts of interest.

## Authors’ contributions

Conceived and designed the project: IZ, KS, PO, PM. Conducted the prospective study: PJ. Performed the retrospective chart review: IZ. Carried out the laboratory experiments: PM, PO, PJ. Analysed the data: IZ, KS, PM. Wrote the paper: IZ. Reviewed the paper: PJ, PM, PO, KS. All authors read and approved the final manuscript.

## Pre-publication history

The pre-publication history for this paper can be accessed here:

http://www.biomedcentral.com/1471-2334/14/155/prepub
